# Plasmids Can Shift Bacterial Morphological Response against Antibiotic Stress

**DOI:** 10.1002/advs.202203260

**Published:** 2022-11-24

**Authors:** Zhigang Yu, Emily C. A. Goodall, Ian R. Henderson, Jianhua Guo

**Affiliations:** ^1^ Australian Centre for Water and Environmental Biotechnology The University of Queensland St. Lucia Brisbane Queensland 4072 Australia; ^2^ Institute for Molecular Bioscience The University of Queensland St. Lucia Brisbane Queensland 4072 Australia

**Keywords:** antibiotic, antibiotic resistance, efflux pump, filamentation, plasmid, toxin‐antitoxin

## Abstract

Bacterial cell filamentation is a morphological change wherein cell division is blocked, which can improve bacterial survival under unfavorable conditions (e.g., antibiotic stress that causes DNA damage). As an extrachromosomal DNA molecule, plasmids can confer additionally advantageous traits including antibiotic resistance on the host. However, little is known about whether plasmids could shift bacterial morphological responses to antibiotic stress. Here, it is reported that plasmid‐free cells, rather than plasmid‐bearing cells, exhibit filamentation and asymmetrical cell division under exposure to sub‐inhibitory concentrations of antibiotics (ciprofloxacin and cephalexin). The underlying mechanism is revealed by investigating DNA damage, cell division inhibitor *sulA*, the SOS response, toxin‐antitoxin module (*parDE*) located on plasmids, and efflux pumps. Significantly higher expression of *sulA* is observed in plasmid‐free cells, compared to plasmid‐bearing cells. Plasmid carriage enables the hosts to suffer less DNA damage, exhibit stronger efflux pump activities, and thus have a higher antibiotic tolerance. These benefits are attributed to the *parDE* module that mediates stress responses from plasmid‐bearing cells and mainly contributes to cell morphological changes. Collectively, the findings demonstrate that plasmids can confer additional innate defenses on the host to antibiotics, thus advancing the understanding of how plasmids affect bacterial evolution in hostile environments.

## Graphical abstract

It is discovered that plasmids can shift bacterial evolutionary response during antibiotic treatment. Plasmids encoding toxin‐antitoxin (TA) system can reverse bacterial filamentation process when exposed to antibiotics, allowing cells to maintain normal growth and suffer less antibiotic‐mediated DNA damage. This morphological shift is also observed in uropathogenic *Escherichia coli* and enables the hosts to exhibit higher antibiotic tolerance.

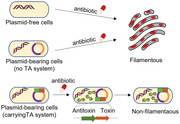

## 1 Introduction

Normal bacterial growth generates daughter cells with a relatively regulated division rate, DNA content, and size.^[^ ^1^ ^]^ Such cell‐size homeostasis has been reported in evolutionarily divergent bacteria^[^ ^2^ ^]^ and metazoan cells.^[^ ^3^ ^]^ However, the cell‐size homeostasis control can be perturbated with stresses that inhibit cell division and cause filamentous bacterial cell morphologies. Filamentation is often considered a “less fit” state of growth, but has been considered as a conserved survival strategy (or “bet‐hedging,” switching from growing to non‐growing states) adopted by bacterial cells against unfavorable conditions, such as antibiotics and the host immune systems.^[^ ^4^ ^]^ This morphological plasticity enables bacterial cells to develop resistance to antibiotics,^[^ ^5^ ^]^ protist predation,^[^ ^6^ ^]^ and phagocytosis by macrophages.^[^ ^7^ ^]^ More seriously, filamentous pathogens can evade these stresses to cause frequent recurrences of common infections,^[^ ^8^ ^]^ for example, urinary tract infections, which affect over 4 million women and cause an economic loss of ≈$1.6 billion annually in the United States.^[^ ^9^ ^]^ Such a morphological phenotype enables filamentous cells, with a much quicker surface turnover, to colonize and spread on abiotic/biotic surfaces in biofilm formation.^[^ ^10^ ^]^ This makes filaments more difficult to be completely cleaned off and will compromise drug therapies. Therefore, it is critical to understand how filamentation proceeds in order to develop strategies to combat bacterial filamentation.

The filamentation phenotype involves delicate processes in response to stress. For example, DNA damage can block cell division and cause bacterial filamentation.^[^ ^11^ ^]^ When DNA damage happens, an inducible DNA repair network known as the SOS response is activated through the binding of *recA* with single stranded DNA and the self‐cleavage of the *lexA* repressor, enabling expression of SOS regulon genes.^[^ ^12^ ^]^ As a result, a cell division inhibitor, *sulA*, blocks FtsZ (a master regulator of bacterial cell division and responsible for the Z ring formation at mid‐cell) polymerization and contributes to non‐septate cellular filamentation.^[^ ^5^ ^]^ Continual exposure to antibiotics (e.g., ciprofloxacin (Cip) and cephalexin (Cep)) inhibits cell growth or kills bacterial cells, while the acquisition of suppressor mutations in genes encoding DNA gyrase (a topoisomerase) and efflux pumps can restore filamentous cells to normal morphology.^[^ ^13^ ^]^ Specifically, when DNA repair is completed or stresses such as antibiotics are removed, *lexA* represses the SOS promoters and the ATP‐dependent Lon protease degrades *sulA*,^[^ ^14^ ^]^ thereby restoring bacterial cell division at mid‐cell with normal size. Altogether, filamentation can be considered an evolutionary pathway for bacteria to survive antibiotic treatment.

As extra‐chromosomal mobile genetic elements, plasmids help the hosts to adaptive evolution and niche expansion in a myriad of environments, thus shifting microbial ecology and evolution.^[^ ^15^ ^]^ Currently, the stable inheritance of plasmids has been ascribed to post‐segregational killing of plasmid‐free cells.^[^ ^16^ ^]^ The plasmid‐encoded genes responsible for such killing encode toxin‐antitoxin (TA) modules, where the stable toxins cannot be neutralized by their labile cognate antitoxins and ultimately cause death among plasmid‐free cells. Such TA modules (e.g., *parDE*) have been found in many plasmids such as RP4 or RK2^[^ ^17^ ^]^ and can protect cells by diminishing bacterial susceptibility to antibiotics such as anti‐gyrase Cip.^[^ ^18^ ^]^ Specifically, the toxin *parE* can also act as DNA gyrase inhibitor to induce bacterial tolerance to antibiotics. Thus, we hypothesize that plasmid‐encoded *parDE* could enable the hosts to evolve in a different pathway from plasmid‐free ones, and to achieve cell size homeostasis even if exposed to antibiotics. However, little is known about whether plasmid‐bearing cells could still develop as filaments under exposure to antibiotics and how the residing plasmids contribute to morphological response from the host bacterium to antibiotic pressure.

Here we aim to address whether and how plasmids shape bacterial morphological evolution under exposure to antibiotics. We used a real‐time microscopy coupled microfluidics to analyze the morphological responses of plasmid‐free and plasmid‐bearing bacterial species (*Escherichia coli* K‐12 MG1655 and *Pseudomonas alloputida*) under exposure to sub‐inhibitory levels of antibiotics (Cip and Cep). Two broad‐host range plasmids RP4 and pKJK5 that encode a *parDE* TA system,^[^ ^17b^ ^]^ as well as a non‐mobile plasmid pWH1266 that does not carry TA modules were employed. We also tested with uropathogenic *E. coli* (UPEC) strains to validate the phenomenon. Cell size was statistically analyzed and the kinetics of filamentation was also measured. A series of *E. coli* J53 strains that carried toxin and antitoxin genes were employed to explore the underlying mechanism, together with phenotypical studies such as cell growth and division, DNA damage, and transcriptional analyses.

## 2 Results

### 2.1 Antibiotics Induce Filamentation in Plasmid‐Free Strains Rather Than Plasmid‐Bearing Strains

**Figure 1:**
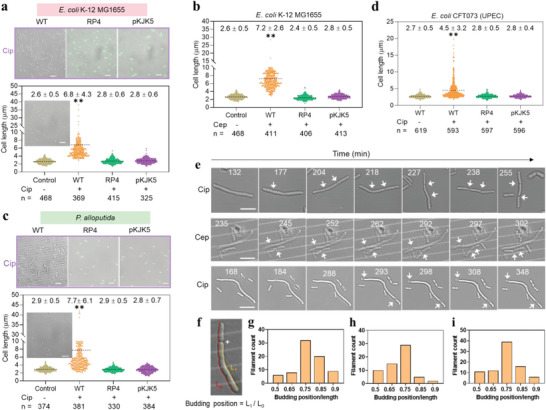
Effect of antibiotics on bacterial cell morphologies. a–d) Statistic analysis of cell length from plasmid‐free/bearing *E. coli* K‐12, *P. alloputida*, and UPEC strains under 2 h exposure to Cip and Cep. The control sample was the bacterial cells without any antibiotic treatment. The analyzed cell number was indicated in the graph. Representative images (a and c) of cell morphology from 0.2× MIC of Cip treated *E. coli* K‐12 strains (plasmid‐free (WT), RP4, and pKJK5) and *P. alloputida* strains (plasmid‐free (WT), RP4, and pKJK5) were presented. The insets in (a and c) were wildtype cell morphology in the absence of antibiotics. (Scale bar, 5 µm). e) Example time‐lapse analyses of budding processes under exposure to antibiotics (upper panel: plasmid‐free *E. coli* K‐12 MG1655 exposed to Cip; middle panel: plasmid‐free *E. coli* K‐12 MG1655 exposed to Cep; bottom panel: plasmid‐free *P. alloputida* exposed to Cip). Images were collected at time points indicated (from starting imaging, the number in each image represents the time in minutes). Arrowheads show budding positions. (Scale bar, 5 µm). f) Determination of relative budding position. g–i) Distribution of budding positions along the filamentous cells (plasmid‐free *E. coli* K‐12 MG1655 exposed to Cip (average length 17.6 ± 3.5 µm, *n* = 61) and Cep (average length 19.0 ± 9.0 µm, *n* = 75), and plasmid‐free *P. alloputida* exposed to Cip (average length 18.5 ± 7.4 µm, *n* = 94), respectively). Data are presented as mean ± SD. Significant differences between the control and the treated groups were tested with Independent‐sample *t*‐test and the Bonferroni correction, * *p* < 0.05 and ** *p* < 0.01.

We first compared the filamentation phenomenon among exponentially growing plasmid‐free and plasmid‐bearing strains (RP4 and pKJK5) under exposure to 0.2× minimum inhibitory concentration (MIC) of antibiotics (Cip and Cep). Images show that plasmid‐free *E. coli* elongated under exposure to 0.2× MIC of both Cip and Cep (**Figure** **1**a,b). Statistical analysis of cell length was also conducted to further confirm the results. Over 300 cells were collected, and their cell length was measured before and after antibiotic treatment. After exposure to 0.2× MIC of antibiotics, the average cell length of all plasmid‐free strains significantly (*p* = 1.5 × 10^−7^) increased. For example, compared to the wildtype cell length (2.6 ± 0.5 µm, the control without antibiotics treatment), the cell length of the plasmid‐free *E. coli* is 6.8 ± 4.3 µm (Figure 1a) and 7.2 ± 2.6 µm (Figure 1b) after 2 h treatment with Cip and Cep, respectively. By contrast, the plasmid‐bearing strains exhibit insignificant (*p* = 0.445) changes in cell length even in the presence of Cip or Cep. For another bacterial species *P. alloputida* under exposure to Cip, the plasmid‐free cells were also elongated because the cell length increased from 2.9 ± 0.5 µm to 7.7 ± 6.1 µm, while the plasmid‐bearing cells still maintained the wildtype cell length (Figure 1c). Such phenomenon was further validated among uropathogenic *E. coli* species (Figure 1d), which can cause urinary tract infection and was isolated from human blood.^[^ ^19^ ^]^ Moreover, we found that less than 50% of cells were filamentous after exposure to antibiotics (Figures S1 and S2, Supporting Information), suggesting that bacterial filamentation is a heterogenous phenomenon.

After that, we investigated the dynamical cell divisions in filamented strains treatment with 0.2× MIC of Cip or Cep using a microfluidic device that allows single‐cell analysis. Time‐lapse imaging reveals that filamentation takes more than 4 h for the wildtype cells (or plasmid‐free) to elongate until the first division. Specifically, filamentous division occurs at a single site, with one division per 60 to 120 min (Figure 1e). Filamentous cells exhibit an asymmetrical budding pattern that occurs repeatably and predominantly at the tips of the filaments (Figure 1f–i), producing short daughter cells that are able to divide normally (Figure 1e). We found that the generated buds of plasmid‐free cells contained chromosome (Figure S3, Supporting Information). It should be noted that it is not clear whether all of the budding events contain bacterial chromosome because filamentation is a heterogenous process.

In addition to 0.2× MIC of Cip and Cep treatments, we also exposed bacterial cells to relatively higher concentrations (0.5× and 0.8×MIC) of the two antibiotics. We found that at such higher levels of antibiotics, bacterial cells that harbor RP4 or pKJK5 plasmid can still maintain normal cell size (Figure S4, Supporting Information). Collectively, exposure to sub‐inhibitory concentrations of antibiotics triggers asymmetrical filamentation of plasmid‐free bacteria, while this phenomenon can be evaded by the plasmid‐bearing bacteria.

### 2.2 Plasmid‐Free Bacteria Exhibit Slower Growth Rate under Exposure to Antibiotics

**Figure 2:**
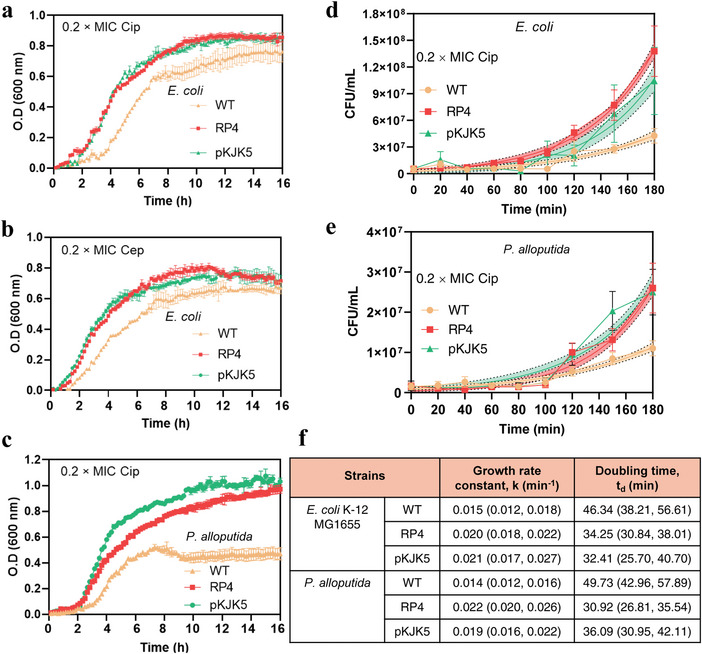
Growth curves of a,b) *E. coli* K‐12 MG1655 strains and c) *P. alloputida* strains under exposure to 0.2× MIC of Cip or Cep (*n* = 3). Plots of the CFU mL^−1^ over time of Cip‐treated cell cultures [d) *E. coli* K‐12 MG1655 strains; e) *P. alloputida* strains] (*n* = 6). All plots were fitted with an exponential growth model. Colour areas represent 95% confidential interval. f) Fitting results of cell growth rate (*k*, min^−1^) and doubling time (*t*_d_, min). Parentheses provide 95% confidence intervals. Data are presented as mean ± SD.

We next questioned whether cell growth of plasmid‐free and plasmid‐bearing cells was arrested following exposure to antibiotics. To test this, the growth curve of each bacterium was plotted by optical density (OD_600_) values over time. No difference of growth between plasmid‐free and plasmid‐bearing bacteria (*E. coli* and *P. alloputida*) was observed in the absence of antibiotics (Figure S5a,b, Supporting Information). In contrast, significant difference was visually observed in the presence of antibiotics (**Figure** **2**a–c). We found that 0.2× MIC of antibiotics visually arrested the growth rate of plasmid‐free strains, other than the plasmid‐bearing strains.

To further reflect this growth arrest, we conducted spot assays of colony number for the groups treated with 0.2× MIC of Cip. The plotted curves were fitted with an exponential growth model to calculate the growth rate (k, min^−1^) and doubling time (t_d_, min) (Figure 2d,e). Consistently, the plasmid‐free cells showed a lower growth rate and a longer doubling time, compared to the corresponding plasmid‐bearing cells (Figure 2f). For example, the average k value of plasmid‐free *E. coli* cell was 0.015 min^−1^, while the k values of RP4‐ and pKJK5‐bearing *E. coli* were 0.02 min^−1^ and 0.021 min^−1^. Consistently, their td values declined from 46.34 min (plasmid‐free *E. coli*) to 34.25 min (RP4‐free *E. coli*) and 32.41 min (pKJK5‐free *E. coli*), respectively. The similar trend was also observed among *P. alloputida* strains. Collectively, plasmid‐free cells exhibit a slower growth rate or cell division than their plasmid‐bearing species under exposure to antibiotics.

### 2.3 A *parDE*‐Type TA System in Plasmid‐Bearing Cells Acts as a Response Module to Antibiotics

A *parDE*‐type TA system has been found in the broad‐host range IncP, IncI and IncF plasmids^[^ ^20^ ^]^ and can serve as a stress response module to antibiotics.^[^ ^18^ ^]^ Given that these plasmid‐bearing cells do not exhibit any filamentation, have a lower doubling time compared to plasmid‐free cells, and carry the plasmid‐encoded *parDE* system, we hypothesized that the *parDE*‐type TA system might have played a role in cell morphological change in plasmid‐bearing cells.

**Figure 3:**
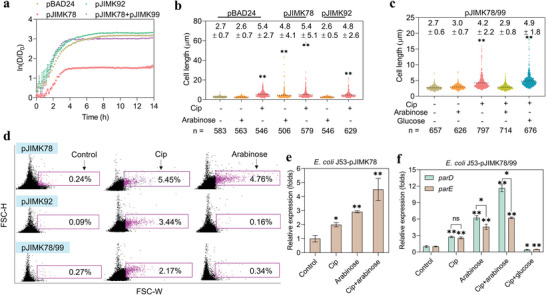
Effect of TA system on cell morphology of *E. coli* J53. a) Bacterial growth of *E. coli* J53 containing different plasmids (pBAD24, pJIMK78, pJIMK92, and pJIMK78/99) in the presence of toxin inducer arabinose. D is the dynamic optical density (OD_600_) of bacterial cells; D_0_ refers to the initial OD_600_ of each sample. b) Cell length of *E. coli* J53 containing different plasmids (pBAD24, pJIMK78, pJIMK92) under exposure to Cip or arabinose, or both. c) Cell length of *E. coli* J53 containing pJIMK78/99 under exposure to Cip, arabinose, or glucose, or their combinations. The dash line in (b and c) means the mean value (in the figures as mean value ± SD) of cell length. d) Representative results of flow cytometry analysis for *E. coli* J53 containing different plasmids (pJIMK78, pJIMK92, and pJIMK78/99) under exposure to Cip and arabinose (*n* = 3). Bacterial cells receiving no treatment were also analyzed as the control group. Fold changes of e) gene *parE* expression in *E. coli* J53 carrying pJIMK78 and f) genes *parDE* expression in *E. coli* J53 carrying pJIMK78/99 under different treatment conditions (*n* = 9). Data are presented as mean ± SD. Significant differences between the control and the treated groups were tested with Independent‐sample *t*‐test and the Bonferroni correction, * *p* < 0.05 and ** *p* < 0.01.

To test this hypothesis, we tested a series of *E. coli* J53 strains that carried toxin and antitoxin relevant genes (*parDE*) under the control of an arabinose‐inducible promoter (Table S1 and Figure S6, Supporting Information). Plasmids pJIMK78 and pJIMK99 were constructed by cloning the *parE* toxin and *parD* antitoxin into pBAD24 and pBAD33 (as expression vectors), respectively;^[^ ^20a^ ^]^ Plasmid pJIMK92 was constructed similar to pJIMK78 but *parE* toxin gene was truncated (C‐terminal, non‐toxic version; Table S1, Supporting Information). Bacterial cells that received both plasmids pJIMK78 and pJIMK99 (pJIMK78/99) can simultaneously express both toxin and antitoxin under arabinose induction. To confirm these features, the growth curves of the above bacteria were measured with induction (0.2% arabinose) of toxin or antitoxin expression. We found that expression of *parE* toxin extended lag phase and arrested growth of *E. coli* J53 carrying pJIMK78 (**Figure** **3**a). The growth of *E. coli* J53 carrying pJIMK78/99 plasmids was not inhibited, which is due to the neutralization between toxin and antitoxin. Expression of the truncated version of *parE* toxin did not inhibit the growth of cell carrying plasmid pJIMK92.

Then, we measured the cell length of these strains following exposure to Cip or TA inducer, or both. The mean cell length of *E. coli* J53 carrying pBAD24 (no *parDE*) was 2.7 ± 0.7 µm without any treatments (Figure 3b; Figure S7, Supporting Information). The bacterial cells with the pBAD24 empty vector control maintained the wildtype cell length under exposure to toxin inducer arabinose, but they elongated (5.4 ± 2.7 µm) when exposed to Cip. Exposure to Cip also enabled *E. coli* J53 carrying pJIMK78, pJIMK92, and pJIMK78/99 plasmids to elongate from the wildtype cell size (Figure 3b,c). Under exposure to TA inducer arabinose, *E. coli* J53 that carried *parE* toxin (pJIMK78) still elongated (4.8 ± 4.1 µm), while *E. coli* J53 that carried either the truncated version of *parE* toxin (pJIMK92) or the full length *parE* toxin in addition to the antitoxin (pJIMK78/99) did not elongate (2.6 ± 0.5 µm and 3.0 ± 0.7 µm). These results suggest that the *parE* toxin induces bacterial filamentation and such activity could be eliminated by the *parD* antitoxin. We further analyzed the cell length of *E. coli* J53 carrying both toxin and antitoxin (pJIMK78/99) by exposing it to the combination of Cip and arabinose, and found that bacterial cells maintained wildtype cell size (2.9 ± 0.8 µm, Figure 3c). Expectedly, the bacterial cells greatly elongated (4.9 ± 1.8 µm) under exposure to Cip and TA repressor glucose (a repressor of the *ara* promoter).

The data suggest that expression of the toxin‐antitoxin system is sufficient to counteract the effect of Cip‐induced cell filamentation. In support of the data, we used flow cytometry to analyze the *E. coli* J53 bacterial cell size with over 200 000 events. For the control group (no Cip or arabinose treatment), very small fractions (< 0.3%) of all bacterial cells were detected in the gating area (larger width (W), means larger time required for cell passing through the channel of detector) (Figure 3d). By contrast, exposure to Cip dramatically increased the fraction of cells detected within this gate. For example, the fraction of *E. coli* J53 carrying *parE* toxin (pJIMK78) increased from 0.24% to 5.45% following treatment with Cip. The treatment with arabinose also caused a dramatic increase (4.76%) of this fraction with larger width. This increase was not observed among *E. coli* J53 that carried either the truncated version of *parE* toxin (pJIMK92) or the full length of *parE* toxin in addition to the antitoxin (pJIMK78/99). Together, these results are consistent with the results from statistical analysis of cell length and further confirm that the toxin‐antitoxin system critically shapes the bacterial morphological response to anti‐gyrase Cip.

Moreover, we quantified the expression of genes *parDE* in *E. coli* J53 carrying toxin (pJIMK78) and both toxin‐antitoxin (pJIMK78/99), under the same treatment conditions. The upregulated expression of *parE* toxin was clearly found among Cip‐ and arabinose‐treated bacterial cells (pJIMK78) (Figure 3e). For example, a 2.0‐fold increase (*p* = 0.015) of *parE* expression was detected following exposure to Cip. Expression of *parE* was further increased by arabinose, evidenced from the largest fold increase (4.5‐folds) by the combination of Cip and arabinose. *E. coli* J53 (pJIMK78/99) also showed the upregulated expression of both *parE* and *parD* (Figure 3f). A 2.8‐ and 2.6‐fold increase of *parD* and *parE* was triggered by Cip. Expression of both genes were continually increased with the treatments of arabinose and the combined Cip and arabinose, under which *parD* showed a larger fold increase than *parE* (*p* = ≈0.009–0.027). By contrast, there was no significant (*p* = 0.666) difference between *parD* and *parE* expression under Cip treatment. Both genes were significantly (*p* = ≈0.005–0.045) downregulated by the treatment of TA repressor (glucose), despite the exposure to Cip.

### 2.4 DNA Damage Is More Severe in Plasmid‐Free Strains than Plasmid‐Bearing Cells

**Figure 4:**
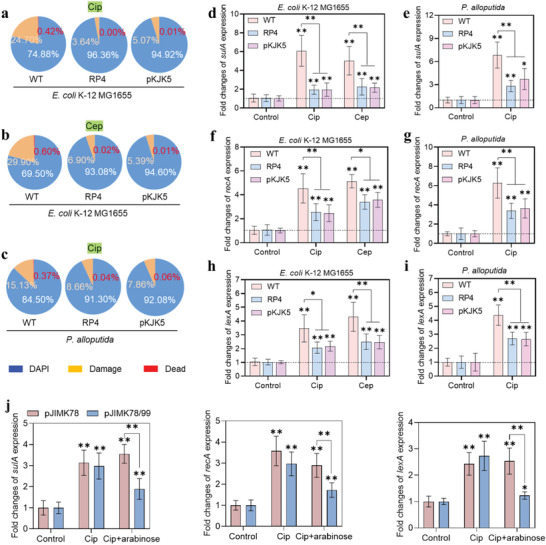
Fractions of DAPI‐ and/or PI‐stained a,b) *E. coli* K‐12 MG1655 strains and c) *P. alloputida* strains under antibiotics (Cip and Cep) treatment. Reverse transcriptional quantitative PCR (RT‐qPCR) analysis of cell division inhibitor *sulA* expression and SOS response related genes (*recA* and *lexA*) expression in d, f, h) *E. coli* K‐12 MG1655 strains and e, g, i) *P. alloputida* strains under antibiotics (Cip and Cep) treatment. j) Expression of SOS response relevant genes (*sulA*, *recA*, and *lexA*) in *E. coli* J53 carrying pJIMK78 or pJIMK78/99 plasmid, in the presence or absence of Cip and arabinose. All samples for PCR quantification were prepared in both biological and technical triplicate (*n* = 9). Data are presented as mean ± SD. Significant differences between the control and the treated groups were tested with Independent‐sample *t*‐test and the Bonferroni correction, * *p* < 0.05 and ** *p* < 0.01.

To confirm whether the DNA damages occurring among antibiotic‐treated cells correlate to cell filamentation, cell variability and fraction of DNA‐containing cells were analyzed by flow cytometry using a two‐colour fluorescence‐based system. Both propidium iodide (PI, staining damaged and dead cells) and 4,6­diamidino­2­phenylindole (DAPI, staining DNA) were used for this analysis. Although a small fraction of cells (plasmid‐free *E. coli*: 0.42% by Cip and 0.60% by Cep, **Figure** **4**a,b; *P. alloputida*: 0.37% by Cip, Figure 4c) were stained by PI after 2 h treatment, it was still higher than that (undetectable) from cells in the absence of antibiotics. Overall, a much smaller fraction of plasmid‐bearing cells stained with PI (*E. coli*‐RP4: 0.01% by Cip and 0.02% by Cep; *E. coli*‐pKJK5: 0.01% by Cip and Cep*; P. alloputida*‐RP4: 0.043% by Cip; *P. alloputida*‐pKJK5: 0.06% by Cip; Figure 4a–c) were observed after 2 h treatment. This suggests that plasmid‐free strains suffer more severe stress than plasmid‐bearing ones during the course of antibiotic treatment.

To validate whether PI‐negative cells are DNA‐free or ‐containing cells, DAPI was used to counterstain the treated cells. In the presence of Cip, 72.08% of plasmid‐free *E. coli* cells were DNA‐containing cells (Figure 4a). By contrast, this fraction increased to 96.74% and 94.60% of RP4‐ and pKJK5‐bearing *E. coli*, respectively. The higher fractions of DAPI‐stained cells were also found among both plasmid‐bearing *E. coli* strains under exposure to Cep (Figure 4b), as well as both plasmid‐bearing *P. alloputida* strains under exposure to Cip (Figure 4c), in comparison with their plasmid‐free strains. In addition, plasmid‐bearing cells suffered less DNA damage (indicated by the fractions of cells stained by both PI and DAPI) than the plasmid‐free cells. Moreover, we found that among *E. coli* J53 strains, the treatment of Cip decreased fraction of DAPI‐stained cell and increased fraction of damaged cell (Figure S8, Supporting Information). Similar to Cip, arabinose also increased cell damage and death in *E. coli* J53 carrying *parE* toxin (pJIMK78). By contrast, arabinose or its combination with Cip did not promote cell damage and death in *E. coli* J53 carrying *parDE* toxin‐antitoxin system (pJIMK78/99). Collectively, these results suggest that even though the majority of treated cells can still maintain their DNA, plasmid‐free strains suffer more severe DNA loss or damage than their plasmid‐bearing ones during antibiotic treatment. Specifically, *parDE* toxin‐antitoxin system confers protection of DNA damage/loss from antibiotic.

To further confirm the difference in DNA damages between plasmid‐free and plasmid‐bearing strains in the presence of antibiotics, the expressions of the SOS response‐related genes (*sulA*, *recA*, and *lexA*) were tracked by quantitative RT‐qPCR. We observed significant increases in the *sulA* gene expression within plasmid‐free cells (*E. coli*: 6.1‐folds by Cip (*p* = 1.2 × 10^−6^) and 5.0‐folds by Cep (*p* = 9.0 × 10^−6^); *P. alloputida*: 6.8‐folds by Cip (*p* = 2.1 × 10^−7^); Figure 4d,e). Such increases in the *sulA* gene expression were also observed among plasmid‐bearing strains, but their fold changes (up to 2.0‐ to 3.7‐folds) were significantly (*p* = ≈0.000018–0.002052) lower than that of their plasmid‐free counterparts. Similarly, the expressions of genes *recA* and *lexA* in plasmid‐bearing cells are significantly (*p* = ≈0.000225–0.024) lower than those found from plasmid‐free ones, under the same conditions (Figure 4f–i). For example, the treatment with Cip induced 2.6‐ and 2.5‐fold increase of *recA* and *lexA* expression in RP4‐bearing *E. coli* cells, while it caused up to 4.5‐ and 3.5‐fold increase in plasmid‐free *E. coli* cells, respectively. These results indicate that plasmid‐free cells are more vulnerable than plasmid‐bearing cells to DNA damage caused by antibiotics. For *E. coli* J53 strains that carry plasmids (pJIMK78 and pJIMK78/99), exposure to Cip also upregulated expression of DNA damage relevant genes (Figure 4j). We also found significant upregulation of these genes expression in *E. coli* J53 carrying pJIMK78/99 when the TA system was induced (by arabinose).

We further confirmed the effect of the TA system on bacterial cell division with TA system‐carrying strains. Again, the treatment of Cip upregulated *sulA* gene expression in all *E. coli* J53 strains (Figure S9a–d, Supporting Information). Such upregulation was also achieved by the treatment of *parE*‐carrying cells (pJIMK78) with TA‐inducer arabinose (Figure S9c, Supporting Information). Although *sulA* gene expression in *parDE*‐carrying cells was upregulated in the combination of Cip and arabinose, the fold increase was significantly lower compared to the same strain treated with Cip (Figure 4j). Meanwhile, the addition of glucose (a repressor of TA expression) induced higher *sulA* gene expression, compared to Cip treatment. Taken together, these results suggest that the TA system results in a less severe SOS response and may protect bacterial cells from antibiotic‐induced DNA damage or cell division arrest.

### 2.5 Plasmid‐Bearing Cells Exhibit Higher Efflux Pump Activity Against Antibiotics

**Figure 5:**
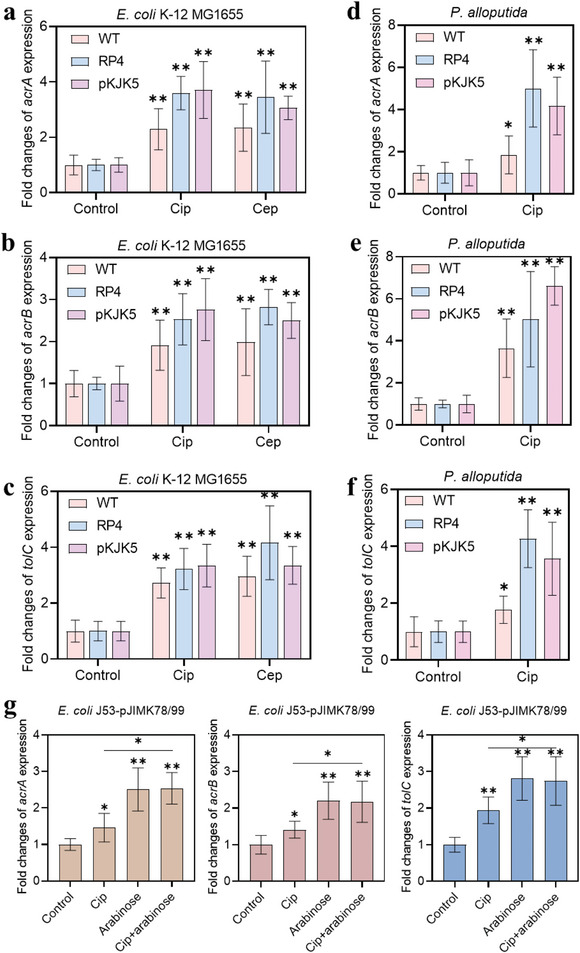
RT‐qPCR analysis of efflux pump genes (*acrA*, *acrB*, and *tolC*) in a–c) *E. coli* and d–f) *P. alloputida* under antibiotic (Cip and Cep) treatment, and in g) *E. coli* J53 carrying plasmid 78/99 under Cip or arabinose treatment (*n* = 9). Data are presented as mean ± SD. Significant differences between the control and the treated groups were tested with Independent‐sample *t*‐test and the Bonferroni correction, * *p* < 0.05 and ** *p* < 0.05.

Gram‐negative bacteria prevent accumulation of antibiotics by overexpressing efflux pumps, and then survive selective pressures. To confirm the activation of efflux pumps in plasmid‐free strains and further explain why plasmid‐bearing strains exhibited higher tolerance to antibiotics than their plasmid‐free ones, we tested the activity of *acrAB*‐*tolC* multidrug efflux pump under different antibiotic treatments. Fold changes in expression of genes such as *acrA*, *acrB*, and *tolC* from both *E. coli* and *P. alloputida* species were quantitatively analyzed by RT‐qPCR technique. Genes encoding efflux pump components were found to be upregulated in antibiotic‐treated plasmid‐free *E. coli* and *P. alloputida* strains compared to the values in untreated cells (**Figure** **5**a–f). For example, up to 2.6‐, 1.7‐, and 2.2‐fold increase of *acrA*, *acrB*, and *tolC* were observed in Cip‐treated plasmid‐free *E. coli* K‐12 MG1655, respectively (Figure 5a–c). This indicates that efflux pumps were activated among plasmid‐free strains under exposure to antibiotics.

Intriguingly, efflux pump activity increased with significantly (*p* = ≈0.00009–0.036) higher fold changes in plasmid‐bearing *E. coli* and *P. alloputida* strains than their plasmid‐free ones. For example, an increase by 3.6‐fold of *acrA*, 3.0‐fold of *acrB*, and 3.3‐fold of *tolC* was observed in Cip‐treated *E. coli* harboring plasmid pKJK5, while only an increase by 2.3‐fold, 1.7‐fold, and 2.2‐fold observed in Cip‐treated *E. coli* wildtype (Figure 5a–c). Similar results were also found in Cep‐treated groups, indicating that the plasmid‐bearing strains exhibit higher levels of efflux pump activity and could then reduce cellular antibiotics to much lower levels than plasmid‐free strains, under exposure to antibiotics. Collectively, these results suggest that expression of efflux pump is higher in plasmid‐bearing cells following exposure to antibiotics, which enables plasmid‐bearing cells to exhibit stronger antibiotic tolerance than their plasmid‐free ones. In the present study, we found that the plasmid (RP4 and pKJK5 plasmids)‐bearing strains showed higher MIC values against antibiotics (Cip and Cep) than the plasmid‐free strains (*E. coli*, *P. alloputida*, and UPEC, Table S2, Supporting Information). For example, compared to plasmid‐free *E. coli* cell, the MIC against Cip increased three and fivefold among RP4‐ and pKJK5‐bearing *E. coli* cells. To further confirm whether the increased antibiotic tolerance was due to plasmid‐encoded efflux pump or the TA module, we further employed *E. coli* J53 with and without carrying *parDE* system. Without arabinose induction, there was no difference of MIC values of *E. coli* J53 strains even if they carried TA relevant genes (Table S2, Supporting Information). In the presence of arabinose, *parDE*‐harboring strain exhibited three times higher in the MIC value toward Cip than that of the original strain (Figure S10, Supporting Information). We also found that induction of TA system dramatically upregulated expression of bacterial efflux pump genes (Figure 5g). These results suggest that the TA system can enhance bacterial efflux pump and increase bacterial tolerance to antibiotics.

## 3 Discussion

**Figure 6:**
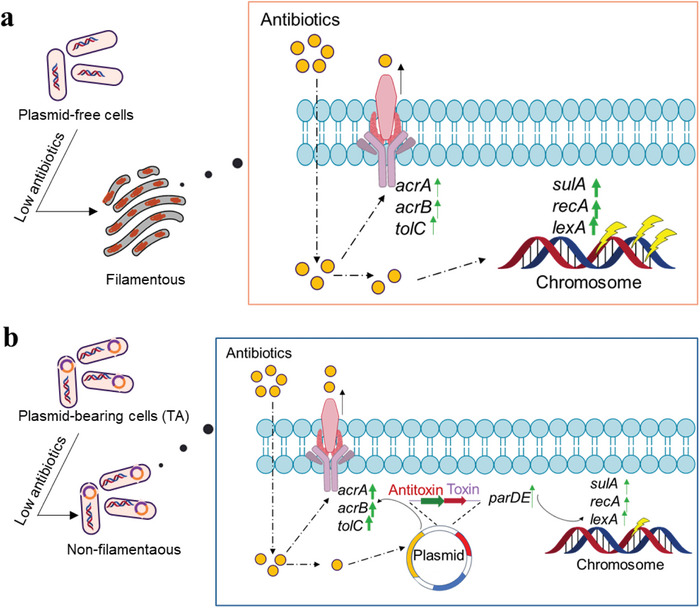
Basic model for morphological response of a) plasmid‐free and b) plasmid‐bearing cells to low concentration of antibiotics. Plasmid‐free cells become filamentous, but plasmid‐bearing cells are not. Under antibiotic treatment, plasmid‐free cells suffer severer DNA damage (e.g., higher expression of genes *sulA*, *recA* and *lexA*), while plasmid‐bearing cells exhibit higher efflux pump activity (e.g., higher expression of genes *acrA*, *acrB*, and *tolC*) and also upregulated toxin‐antitoxin (*parDE*) expression. Relative higher gene expression is indicated by the wider arrows.

Currently, filamentation has been considered as one of many general survival strategies of bacteria in response to antibiotics, with the cell volume continually growing yet cell division arrested.^[^ ^21^ ^]^ Antibiotics such as Cip and Cep have been reported to cause bacterial filamentation among plasmid‐free strains,^[^ ^22^ ^]^ due to the activated SOS response or chromosomal DNA damage by antibiotics. As chromosome‐independent genome, plasmids can diversify the host genetics and especially provide the hosts with additional traits, aside from antibiotic resistance. The plasmids can be well maintained by the host because of their post‐segregational killing or addiction systems that encode par toxin‐antitoxin (TA) module,^[^ ^16^, ^23^ ^]^ which can be conjugated to a wide range of bacterial cells. It has been reported that TA systems play roles in bacterial stress responses and antibiotic tolerance,^[^ ^24^ ^]^ but whether they could shift bacterial morphological response to antibiotics is not clear yet. Here, for the first time we report that the plasmids‐mediated TA systems can shift bacterial morphological response to antibiotics. Specifically, plasmid‐carried *parDE*‐type TA system helps the hosts maintain wildtype cell size (compared with filamentation in plasmid‐free and TA‐free cells) and suffer less DNA damage, under exposure to sub‐MIC of antibiotics (**Figure** **6**). In addition, plasmid‐encoded efflux pump enables the host to be more tolerant to antibiotics, thus alleviating the filamentation process.

Similar to Cip that targets bacterial DNA gyrase and directly causes DNA damage, other DNA‐damage agents such as UV irradiation and mitomycin‐C can also induce asymmetric filamentation.^[^ ^25^ ^]^ These DNA damage and morphological changes can be reversed by plasmid carriage. Our data presented here show that during exposure to sub‐MIC of antibiotics (Cip and Cep), plasmid‐free cells, rather than plasmid (RP4/pKJK6)‐bearing cells, exhibit dynamically asymmetrical budding pattern that preferentially occurs at the tip of cell (Figure 1). Such phenomenon is further validated with UPEC strains containing RP4, pKJK5, or not. The generated offspring maintain normal cell size and growth dynamics,^[^ ^25a^ ^]^ and potentially evolve as antibiotic‐resistant mutants.^[^ ^26^ ^]^ Filamentous plasmid‐free cells suffer more severe DNA damage than plasmid‐bearing cells in response to antibiotics (Figure 4a,b). This finding is also supported by the expression of cell division inhibitor *sulA* and SOS response regulators *recA* and *lexA* (Figure 4c–h). However, for plasmids such as pWH1266 that does not carry a TA system and can be easily lost,^[^ ^27^ ^]^ the plasmid‐bearing cells also showed asymmetric filamentation under exposure to antibiotics (Figure S11, Supporting Information). Given that conjugative plasmids (RP4 and pKJK5) used in this study carry a plasmid maintenance system (i.e., the toxin‐antitoxin *parDE* module) (Figure S12, Supporting Information) and bacterial efflux pumps, we propose that the *parDE* system and efflux pump could modulate bacterial morphological response to antibiotics. It has been reported that filamentous cells have division site selection and size control, which are regulated by spatiotemporal oscillations of Min and Fts dynamics.^[^ ^2^, ^28^ ^]^ During such dynamic process, bacterial cells continuously monitor absolute length to control cell size and DNA segregation. Further studies about whether and how plasmids could interact with the Min and Fts systems will comprehensively advance our understanding of the roles of plasmids in bacterial evolution.

Exposure to subinhibitory levels of antibiotics can give rise to bacterial persisters, which filament extensively and suffer impressive DNA damage as well as the ensuing SOS response.^[^ ^29^ ^]^ Gene pairs, known as TA systems, can also regulate bacterial persistence and dormancy.^[^ ^30^ ^]^ TA gene loci are abundant in bacterial chromosomes, phages, and in particular plasmids that can offer extra adaptive traits to the hosts. It has been reported that plasmids can drive bacterial evolution of antibiotic resistance.^[^ ^31^ ^]^ However, little is known about whether plasmids could shape bacterial morphological response to antibiotics. Our results confirm that the carriage of the *parDE* module can alleviate antibiotic‐mediated DNA damage (Figure 4j, Figures S8 and S9, Supporting Information) and finally revert the filamentous cell to wildtype size (Figure 3). This suggests that the *parDE* module provides evolutionary niche adaptation for bacteria to antibiotic treatment. For example, activation of *parDE* module increases up to 1000‐fold bacterial survival of antibiotics that target on bacterial normal division and cell membrane synthesis.^[^ ^20a^ ^]^ Given that bacteria are exposed to non‐lethal levels of antibiotics in the environment and human urine (up to µg L^−1^),^[^ ^32^ ^]^ our results have evolutionary implications of both environmental and clinical aspects with a large niche space (sub‐inhibitory levels of antibiotics). More TA systems should be considered to generalize such results in further studies. Conditions where bacterial SOS response (e.g., *recA* activity) is compromised needs to be considered to further confirm the effects of TA system on bacterial DNA damage and the SOS response. For example, tests with ∆*sulA* strains can be conducted to strengthen our findings.

We also found that plasmids such as RP4 and pKJK5 encoding efflux pump genes enable their hosts to tolerate higher concentrations of antibiotics. This is consistent with previous studies that plasmid‐encoded multidrug efflux pump confers antibiotic resistance in the host.^[^ ^33^ ^]^ Given that the tested plasmids also carry TA system, it is not clear whether the increased antibiotic tolerance is due to plasmid‐encoded efflux pump or other contributors. In the present study, we used plasmids like pBAD‐based pJIMK78 and pJIMK99 (encoding no efflux pump) and found that plasmid carriage fails to enhance bacterial susceptibility to antibiotics (Table S2, Supporting Information). Intriguingly, exposure to arabinose (induction of TA system) reduces bacterial susceptibility (threefold increase in the MIC value) to Cip (Figure S10, Supporting Information). This is further supported by the upregulated expression of efflux pump relevant genes (Figure 5g). Altogether, these results suggest that the TA system could stimulate bacterial efflux pump activity and increase bacterial tolerance to antibiotics. More molecular work about how TA system could modulate bacterial efflux pump activity is needed.

Collectively, our study for the first time unravels the roles of plasmids in physiological responses of bacteria to antibiotic treatment and in particular how the plasmid‐encoded TA system shapes bacterial evolution under antibiotic selective pressure. Filamentation is a conserved phenotypical strategy that enables pathogens such as UPEC to subvert host innate defenses and consequently leads to recurrent urinary tract infections. In addition to antibiotics, biofilm formation and protist predation can also be associated with bacterial filamentation as a survival strategy. More studies are needed to reveal whether and how antibiotic‐induced filamentation could impact biofilm formation and protist predation. Although our work show that plasmid‐encoded TA module can help bacterial cells maintain wildtype size under exposure to antibiotics, it does not mean that plasmid‐carrying UPEC strains could be eradicated by the host immune defenses or cause low risks of infections. Instead, the plasmid‐bearing cells could survive innate immune response via other pathways, such as enhancing the colonization and biofilm formation in urinary tract.^[^ ^34^ ^]^ Future studies should test more TA systems or employ UPEC strains with various TA systems to further generalize such results in clinic settings. It should be noted that TA systems are generally abundant in the major human pathogens and environmental distinct species, and can regulate bacterial pathogenesis.^[^ ^35^ ^]^ Further studies including characterization of putative plasmid‐harboring cell signals and understanding of bacterial TA regulation will enable us to predict bacterial evolution under selective pressure and the pathogenesis of bacterial infections and will also oblige us to develop effective mitigation of the relevant infections.

## 4 Experimental Section

### Bacterial Strains, Plasmids, and Growth Conditions

All bacterial strains (*E. coli* K‐12 MG1655, *E. coli* J53 and *P. alloputida* strains, as well as uropathogenic *E. coli* strains) and the plasmids used in this study are presented in Table S1, Supporting Information. *P. alloputida* strains were also used as representatives of environmentally relevant species. The 60 Kbp RP4 plasmid carries resistance to ampicillin, kanamycin and tetracycline,^[^ ^36^ ^]^ while the 54 Kbp pKJK5 plasmid, originally isolated from a soil/manure environment, carries resistance to tetracycline and trimethoprim.^[^ ^37^ ^]^ Both plasmids harbor a class I integron (PA10403‐*gfpmut3*) that encodes *gfp*.^[^ ^38^ ^]^ The culture conditions of the bacteria are shown in Text S1, Supporting Information.

### Minimum Inhibitory Concentration (MIC) Determination

In this study, the MIC is defined as the lowest concentration of antibiotic (Cip), a DNA gyrase and topoisomerase inhibitor; Cep, a peptidoglycan synthesis inhibitor) that inhibits 90% of bacterial growth. For each strain, the overnight culture was initially diluted 1000 times with fresh LB medium (Miller). A total volume of 150 µL LB medium that contains cells (≈10^5^ CFU mL^−1^) and antibiotics (Cip: 0, 0.005, 0.01, 0.02, 0.05, 0.1, 0.2, and 0.5 mg L^−1^; Cep: 0, 5, 10, 12.5, 20, 22.5, 25, and 50 mg L^−1^) were loaded onto 96‐well flat‐bottom plates. End‐point optical density at a wavelength of 600 nm (OD_600_) was recorded by a CLARIOstar Multimode plate reader (BMG LABTECH). Each sample was prepared in biological triplicate. The MICs were summarized in Table S2, Supporting Information.

### Cell Morphology Analysis and Growth Assays

Induction of cell morphology change was performed on cultures grown in LB medium at 30 °C with Cip or Cep treatment during 120 min. Overnight culture of each strain was sub‐cultured at 1:100 dilution and grown to mid‐exponential phase (OD_600_ of ≈0.2). After that, either Cip or Cep was added at a sub‐MIC final concentration of 0.2× MIC. Meanwhile, the effects of TA induction or repression (arabinose and glucose, respectively) on the morphology of exponential growing *E. coli* J53 species (with different plasmids that carried TA genes) were also evaluated. The arabinose or glucose was added to the samples at a final concentration of 0.2%. After 120 min, the treated or untreated cell cultures were collected and 10 µL of each culture were pipetted onto 1% agarose‐padded slides, which was covered with a glass coverslip. After that, cell morphology was imaged by an inverted laser scanning confocal microscope (ZEISS LSM 710, AxioObserver) that was equipped with Plan‐Apochromat 40×/1.4 Oil DIC M27 objective and 3‐channel QUASA spectral PMT array. Acquisition was set up with 0.39 µs pixel time of both green and red fluorescence using a Fluo LED Spectra X light source. Green fluorescence was detected at 488 nm excitation wavelength and 518 nm emission wavelength. Snapshot or time‐lapse images were collected with 1024 × 1024 pixels per image. Cell length was measured by a freeware Fiji with the MicrobeJ plugin and was plotted on GraphPad Prism software 8.0.

To investigate the effects of different treatments (antibiotics or TA inducers) on bacterial growth, the growth curves were plotted as the OD600 value recorded every 5 min for 16 h of incubation at 30 °C (*P. alloputida*) or 37 °C (*E. coli* and UPEC) with shaking. In this study, Cep was not employed for the treatment of *P. alloputida* strains because of their high tolerance (>1000 mg L^−1^ of Cep, in terms of MIC measurement). To calculate cell division time, the CFU number was enumerated by plate culturing. After sub‐culturing and growth to the exponential phase (OD_600_ of ≈0.2), all cultures were treated with 0.2× MIC of Cip and were incubated with shaking (120 rpm) at 30 °C. Samples were collected at the indicated times, serially diluted, and spread onto LB agar plates. All plates were incubated 48 h at 30 °C or 37 °C before enumerating CFU mL^−1^. All samples were prepared in biological triplicate and were measured in technical duplicate.

### Time‐Lapse Imaging of Cell Division and Filamentation

The budding process of filamentous bacterial cells was dynamically visualized by LSM710. After sub‐culturing in LB media and growth to mid‐exponential phase, 0.2× MIC of Cip or Cep was added to the cultures (plasmid‐free *E. coli* K‐12 MG1655 and *P. alloputida*). Afterward, 10 µL of each culture was pipetted onto 1% agarose‐padded slide and was covered with a glass coverslip. The slide was loaded onto LSM710 for real‐time visualization. In addition, nucleoids in the treated cells were also visualized by following the same procedures, but the cultures were stained with DAPI before treatment with Cip.

### Flow Cytometry Analysis

Overnight cultures were diluted 1:1000 in fresh LB medium and incubated for 90 min. After that, the cultures with the same concentration were treated by 0.2× MIC of Cip or Cep for 2 h. Samples of treated and untreated cultures were then stained with propidium iodide (PI, final concentration of 10 mg L^−1^) and DAPI (final concentration of 10 mg L^−1^) at room temperature (25 ± 2 °C) for 20 min in the dark. Cells from each sample were analyzed by a CytoFLEX S flow cytometer (Beckman Coulter, USA). Analysis was conducted on the cytometer with a yellow laser (561 nm) and a 585/42 emission filter for PI and with a violet laser (405 nm, *mCherry*) and a 610/20 of excitation wavelength. The unstained and heated (80 °C, 2 h) bacterial cells were simultaneously prepared as controls for the intact (live or DNA‐containing cells) and dead or damaged cells, respectively. The fractions of DAPI‐stained cells, damaged or dead cells were defined as the percentage of stained cells divided by the total events (at least 40 000 events detected for each sample).

The flow cytometer was also used to further confirm cell morphology changes with much more bacterial events. The normal bacterial size was calibrated with small beads (≈3 µm). All samples were analyzed based on two gates, FSC‐H and FSC‐A, to exclude doublets. Here, tested was conducted with a series of *E. coli* J53 species that contained toxin (pJIMK78) or truncated toxin (pJIMK92), or both toxin and antitoxin (pJIMK78/99). Samples of bacterial cells without antibiotics or inducers treatment were also analyzed. A total of at least 200 000 events were acquired for each sample, in biological triplicate.

### RNA Extraction and Reverse Transcriptase Assay

Expression of genes responsible for SOS response (*sulA*, *recA*, *lexA*), TA system (*parD* and *parE*), and efflux pump (*acrAB*, and *tolC*) were measured by reverse‐transcriptase quantitative PCR (RT‐qPCR). Cell suspensions treated with antibiotics, 0.2% arabinose or 0.2% glucose were the treated group, while those without any chemical treatment were the control group. After 2 h, cell pellets were collected by centrifuging at 4 °C, 6000 rpm for 6 min. The total RNA was then extracted using the RNeasy Mini Kit (QIAGEN, Germany) according to the manufacturer's instructions, with an extra cell lysis step of beads‐beating. The extracted RNA was treated with DNase (TURBO DNA‐free Kit, Ambion) to avoid DNA contamination (confirmed by PCR). The RNA concentration was quantitatively measured with Qubit RNA HS assay and Qubit 3.0 fluorometer (Invitrogen, Carlsbad, California, USA). After that, 10 µg of RNA from each sample were used for cDNA clone by high‐capacity cDNA reverse transcriptase kit (Applied Biosystems). One microliter of cDNA was used for PCR (primers shown in Table S3, Supporting Information). All reactions were performed on 384‐well plates using a QuantStudio 6 Flex real‐time PCR system (Applied Biosystems). The total volume of 10 µL was used for all reactions. Detailed information about PCR programs were shown in Table S4, Supporting Information. The specificity of all primers for qPCR assays was performed by cloning of PCR products from selected positive samples in this study (Table S5, Supporting Information). A bacterial 16S rRNA gene (BACT1369F‐PROK) was used as a reference gene.^[^ ^39^ ^]^ The stability of 16S rRNA gene expression was evaluated by the variation of threshold cycle (Ct) number from treated and untreated samples. The relative expression of all genes was calculated by the 2^−∆∆Ct^ (Livak) method^[^ ^40^ ^]^ and was normalized to the geometric mean of 16S rRNA ∆Ct values. All samples were analyzed in biological triplicates and technical triplicates.

### Statistical Analysis

Data were presented as the mean ± standard deviation (SD) in the graph. All results were analyzed by Analysis of variance (ANOVA) and Independent‐sample *t*‐test through SPSS 27.0 (SPSS, Chicago, USA). The calculated *p* values were further corrected by the Bonferroni method.^[^ ^41^ ^]^ A value of *p* < 0.05 was considered to be statistically significant, while a value of *p* < 0.01 was considered to be highly significant.

## Conflict of Interest

The authors declare no conflict of interest.

## Author Contributions

Z.Y. conceived, performed the experiments, analyzed data, interpreted the results, and wrote manuscript. E.C.A.G. and I.R.H. assisted with data interpretation and manuscript revisions. J.G. conceived and designed the research, and assisted with data interpretation and manuscript writing.

## Supporting information

Supporting Information

## Data Availability

The data that support the findings of this study are available from the corresponding author upon reasonable request.
