# Supplementary material for: Plasmids Can Shift Bacterial Morphological Response against Antibiotic Stress
Source: Adv Sci (Weinh). 2022 Nov 24;10(2):2203260. doi: 10.1002/advs.202203260 (PMC9839882; doi:10.1002/advs.202203260)
Supplement: Supplementary file 1 — Supporting Information [file ADVS-10-2203260-s001.pdf]

ADVANCED SCIENCE
Open Access

Supporting Information

for *Adv. Sci.*, DOI 10.1002/advs.202203260

Plasmids Can Shift Bacterial Morphological Response against Antibiotic Stress

*Zhigang Yu, Emily C. A. Goodall, Ian R. Henderson and Jianhua Guo**

Supplementary Information

Plasmids can shift bacterial morphological response against antibiotic stress

*Zhigang Yu, Emily C. A. Goodall, Ian R. Henderson, Jianhua Guo**

Z. Yu, J. Guo
Australian Centre for Water and Environmental Biotechnology, The University of Queensland, St. Lucia, Brisbane, QLD 4072, Australia
E-mail: jianhua.guo@uq.edu.au

E. C. A. Goodall, I. R. Henderson
Institute for Molecular Bioscience, The University of Queensland, St. Lucia, Brisbane, QLD 4072, Australia.

This file contains

- Text S1
- Tables S1 to S5
- Figures S1 to S12
- References

Text S1. Bacteria culture conditions

Plasmid-free *Escherichia coli* strain K-12 MG1655 and *Pseudomonas alloputida* strain were separately grown overnight at 37 ºC and 30 ºC in Miller LB media. RP4-bearing *E. coli* and *P. alloputida* strains were cultured in LB that contained 100 mg/L ampicillin, while pKJK5-bearing *E. coli* and *P. alloputida* strains were cultured in LB that contained 100 mg/L kanamycin. Overnight cultured cells were sub-cultured in fresh LB media for another 2 h with 120 rpm shaking. Afterwards, cells were collected by centrifugation (5000 × g, 5 min) and were used for further use.

Table S1. Bacterial strains and plasmids used in this work

	**Genotype/description**	**Source**
**Strains**		
MG1655/wild type	*E. coli* K-12 MG1655	This study
MG1655/*gfp*-RP4	*E. coli* K-12 MG1655 carrying RP4 plasmid	This study
MG1655/*gfp*-pKJK5	*E. coli* K-12 MG1655 carrying pKJK5 plasmid	This study
KT2440/wild type	*P. alloputida*	This study
KT2440/*gfp*-RP4	*P. alloputida* carrying RP4 plasmid	[1]
KT2440/*gfp*-pKJK5	*P. alloputida* carrying pKJK5 plasmid	This study
UPEC CFT073	Uropathogenic *E. coli* CFT073	
UPEC CFT073 mutant	Uropathogenic *E. coli* CFT073 with resistance to chloramphenicol	This study
UPEC CFT073/RP4	Uropathogenic *E. coli* CFT073 (with resistance to chloramphenicol) carrying RP4 plasmid	This study
UPEC CFT073/pKJK5	Uropathogenic *E. coli* CFT073 (with resistance to chloramphenicol) carrying pKJK5 plasmid	This study
J53/wild type	*E. coli* J53 without plasmid	[1]
J53/ pBAD24	*E. coli* J53 with expression vector that contains arabinose inducible promoter (Amp^R, pBR322 replicon)	[2]
J53/ pJIMK78	*E. coli* J53 with pJIMK78 plasmid that contains *parE* toxin under an arabinose inducible promoter. The *parE* toxin was amplified from pJIE512b and was cloned into EcoRI and XbaI sites of pBAD24 plasmid.	[2]
J53/ pJIMK92	*E. coli* J53 with pJIMK92 plasmid that contains the C-terminus truncated *parE* toxin. The *parE* toxin was amplified from pJIE512b and was cloned into *EcoRI* and	[2]

	XbaI sites of pBAD24 plasmid.	
J53/ pJIMK78+99	*E. coli* J53 with both pJIMK78 and pJIMK99 plasmids. pJIMK99 contains *parD* antitoxin that was amplified from pJIE512b and was cloned into *EcoRI* and *XbaI* sites of pBAD33 (carrying resistance to gentamycin, Gem^R) plasmid under an arabinose inducible promoter.	[2]
**Plasmids**		
gfp-RP4	IncP-α plasmid is labelled with *gfp* gene and contains resistance genes ($ampicillin^R$, $kanamycin^R$, and $tetracycline^R$)	This study
gfp-pKJK5	IncP-α plasmid is labelled with *gfp* gene and contains resistance genes ($kanamycin^R$ and $trimethoprim^R$)	This study
pBAD24	Expression vector that contains arabinose inducible promoter (Amp^R, pBR322 replicon)	[2]
pJIMK78	Contains *parE* toxin under an arabinose inducible promoter. The *parE* toxin was amplified from pJIE512b and was cloned into EcoRI and XbaI sites of pBAD24 plasmid.	[2]
pJIMK92	Contains the C-terminus truncated *parE* toxin. The *parE* toxin was amplified from pJIE512b and was cloned into *EcoRI* and *XbaI* sites of pBAD24 plasmid.	[2]
pJIMK99	contains *parD* antitoxin that was amplified from pJIE512b and was cloned into *EcoRI* and *XbaI* sites of pBAD33 plasmid (Gem^R) under an arabinose inducible promoter.	[2]

Table S2. MICs of different bacterial species against Cip and Cep

Strains	MIC	
	**Cip, μg/L**	**Cep, mg/L**
Plasmid-free *E. coli* K-12	20	12.5
RP4-bearing *E. coli* K-12	60	25
pKJK5-bearing *E. coli* K-12	100	22.5
Plasmid-free *P. alloputida*	50	>1000
RP4-bearing *P. alloputida*	60	>1000
pKJK5-bearing *P. alloputida*	60	>1000
Plasmid-free UPEC	128	-
RP4-bearing UPEC	256	-
pKJK5-bearing UPEC	256	-
Plasmid-free *E. coli* J53	32	-
pBAD24-bearing *E. coli* J53	32	-
pJIMK78-bearing *E. coli* J53	32	-
pJIMK92-bearing *E. coli* J53	32	-
pJIMK99-bearing *E. coli* J53	32	-
pJIMK78/99-bearing *E. coli* J53	32	-

Note: Each sample was prepared at least in biological triplicate; “-” means no measurement.

Table S3. PCR primers used in this study

**Primer**		**Sequence (5' to 3')**	**Primer length (bp)**	**References**
parD	F	gcgaattcTAGTAATGACGAGGTGATAA		[2]
	R	gctctagaTCATTTACCGGCAACCTTCCT		
parE	F	ctgaattcAAGGTTGCCGGTAAATGATG		
	R	gctctagaAAATGCGGGTGAATAACCA		
sulA	F	CGTCAACGGTACCGCTGTAACTG	23	[3]
	R	GCCTGAAGTGAGCTCAATCAATCC		
acrA	F	AGCCCTAACAGGATGTGACG	20	[4]
	R	GCTTCGATGTCGCTACCTTC		
acrB	F	GATTACCATGCGTGCAACAC	20	[4]
	R	TCTGCAAGCAACTGGTTACG		
tolC	F	CTGAAAGAAGCCGAAAAACG	20	[4]
	R	CTGGCCCATATTGCTATCGT		
recA	F	TCCGGTAAAACCACGCTGAC	20	[2]
	R	CGTGCGTAGATTGGGTCCAG		
lexA	F	CGCGGCTGAAGAACATCTGA	20	[2]
	R	GCGGCAACCCTTCTTCCTCT		
16S rRNA	F	CGGTGAATACGTTCYCGG		[5]
	R	GGWTACCTTGTTACGACTT		

Table S4. The optimized RT-qPCR programs

**Target genes**	**Stage**	**Condition**
sulA, *recA*, *lexA*<br><br>*acrA*, *acrB*, *tolC*	Hold stage	95 ℃, 2 min
	PCR stage	95 ℃, 5 s;
		55 ℃, 20 s;
		60 ℃, 20 s;
		40 cycles
	Melt curve stage	95 ℃, 15 s
		60 ℃, 1 min
		95 ℃, 15 s
parD, *parE*	Hold stage	95 ℃, 3 min
	PCR stage	95 ℃, 30 s;
		55 ℃, 30 s;
		72 ℃, 1 min
		40 cycles
	Melt curve stage	72 ℃, 5 min

Table S5. Amplification efficiency of primers tested with RT-qPCR

**Gene**	**Slope**	**Efficiency**
parD	3.2921	101.26
parE	3.3018	99.799
sulA	3.2867	101.4914
tolC	3.3806	97.60843
recA	3.2224	104.3279
lexA	3.2633	102.5062
acrA	3.3308	99.63109
acrB	3.2179	104.5322
PROK	3.414	96.29604

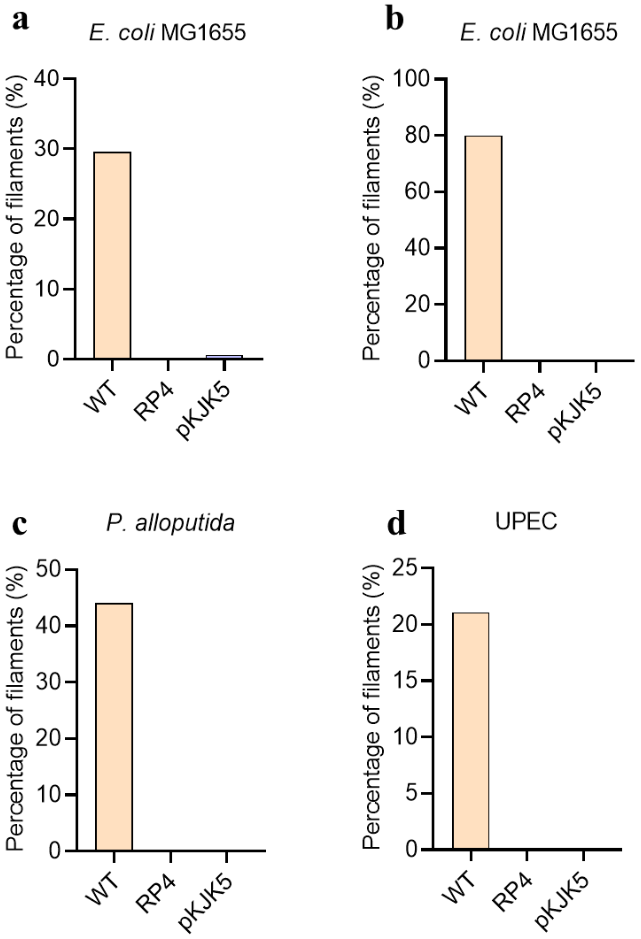

Figure S1. Percentage of filaments from (a) Cip-treated *E. coli* MG1655, (b) Cep-treated *E. coli* MG1655, (c) Cip-treated *P. alloputida*, and (d) Cip-treated UPEC

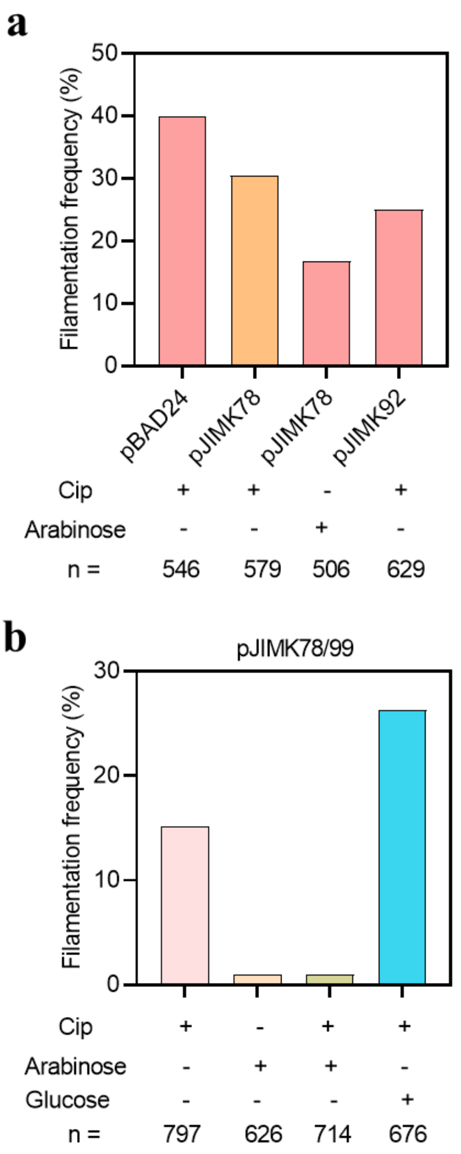

Figure S2. Filamentation frequency of *E. coli* J53 strains containing (a) plasmids pBAD24, pJIMK78, pJIMK92, and pJIMK78/99, and (b) plasmid pJIMK78/99

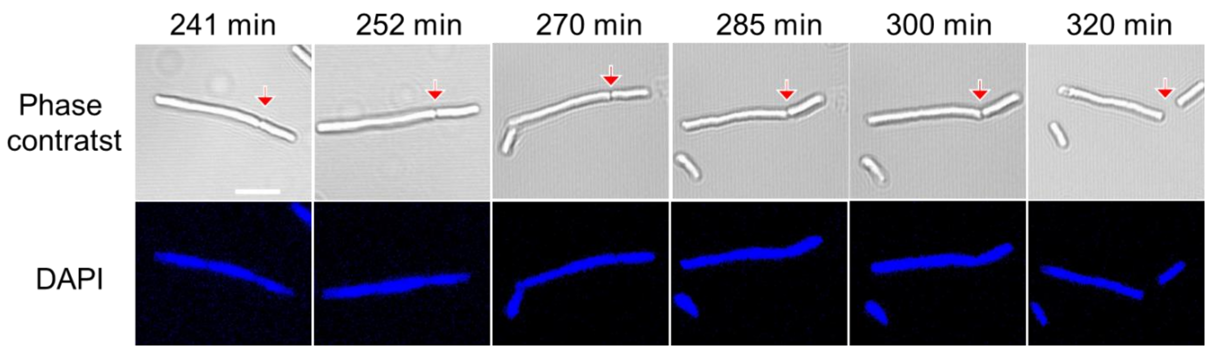

Figure S3. Time-course DAPI staining analysis of plasmid-free *E. coli* K-12 MG1655 strain under exposure to Cip. Arrowhead indicates the position where bacteria divided.

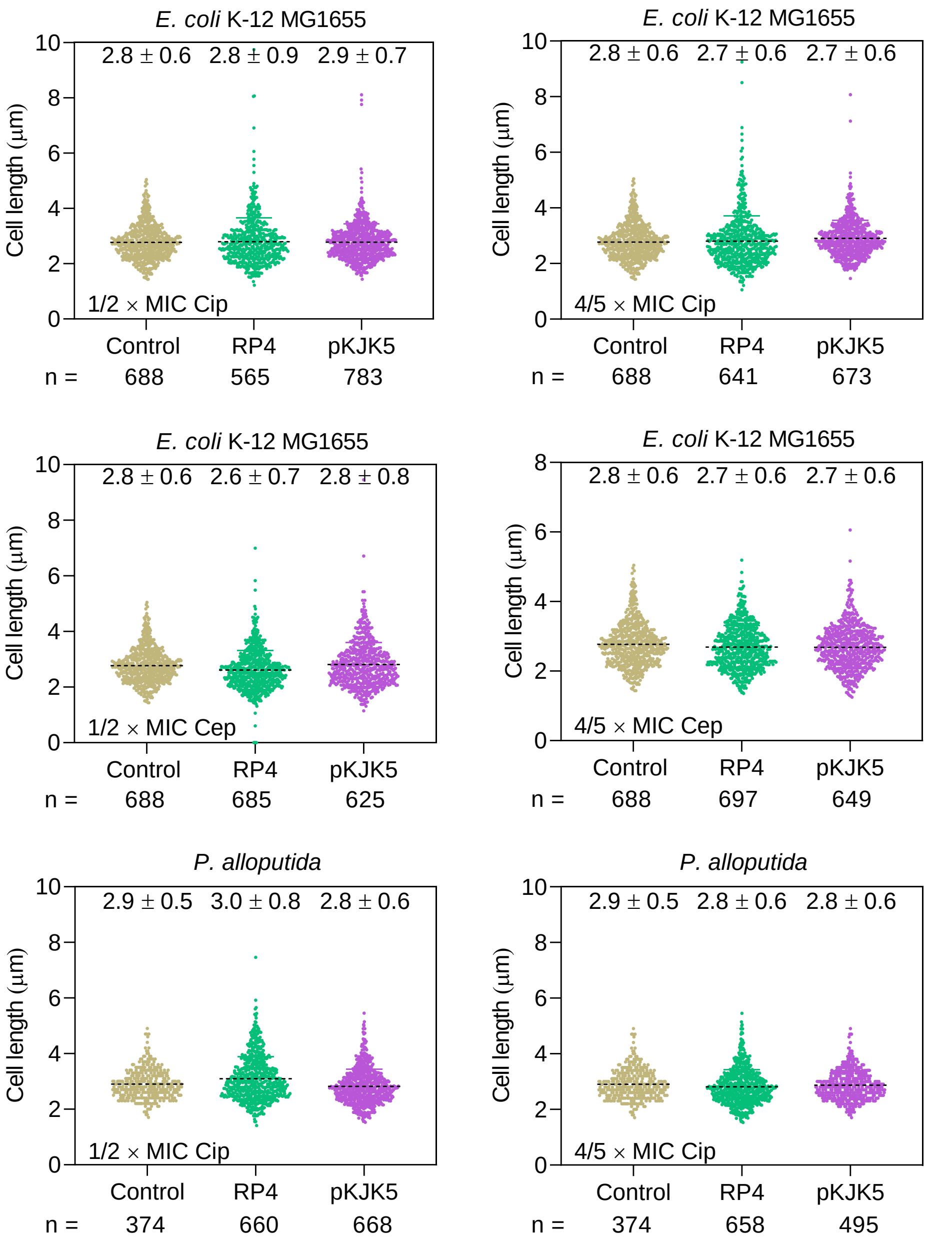

Figure S4. Effect of higher dosages (0.5 × and 0.8 ×MIC) of antibiotics on bacterial cell length. The control sample was the bacterial cells without any antibiotic treatment. The analyzed cell number was indicated in the graph.

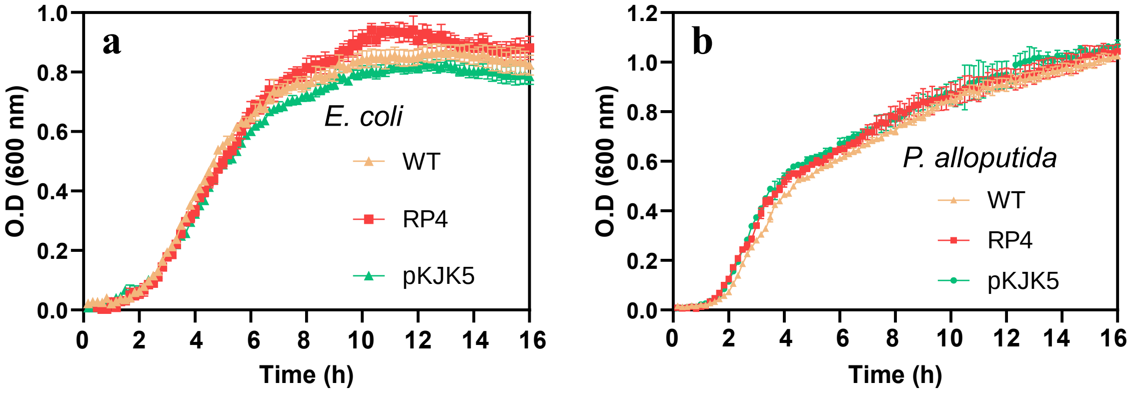

Figure S5. Growth curves of strains in the absence of antibiotics. **a**, Plasmid-free and plasmid-bearing *E. coli* K-12 MG1655 strains in the absence of antibiotics. **b**, Plasmid-free and plasmid-bearing *P. alloputida* strains in the absence of antibiotics.

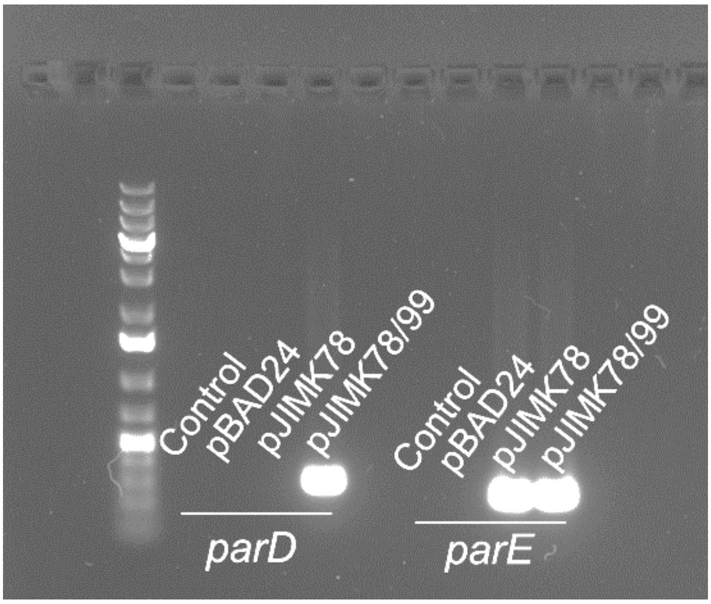

Figure S6. Gel imaging of *parE* toxin and *parD* antitoxin from *E. coli* J53 strains that carried pBAD24, pJIMK78, and pJIMK78/99 plasmids. The control refers to the reagents used for DNA extraction.

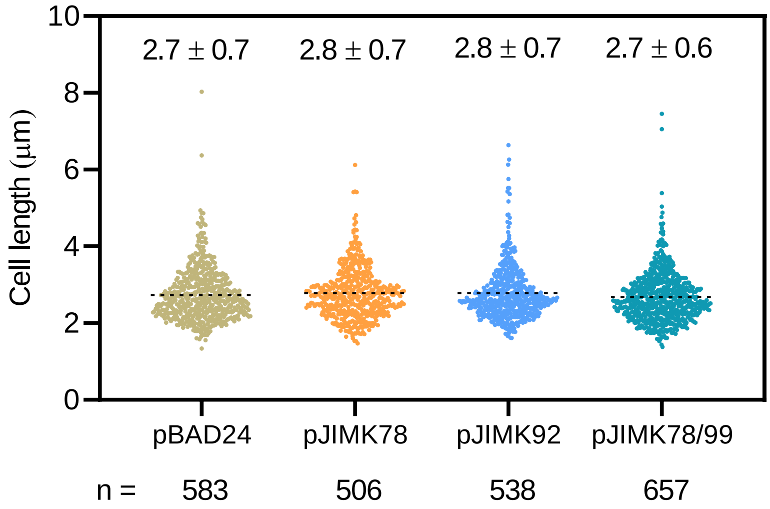

Figure S7. Cell length of *E. coli* J53 that carried different plasmids (pBAD24, pJIMK78, pJIMK92, and pJIMK78/99) without antibiotics or TA inducer treatment. The dash line in (**b** and **c**) means the mean value (in the figures as mean value ± SD) of cell length.

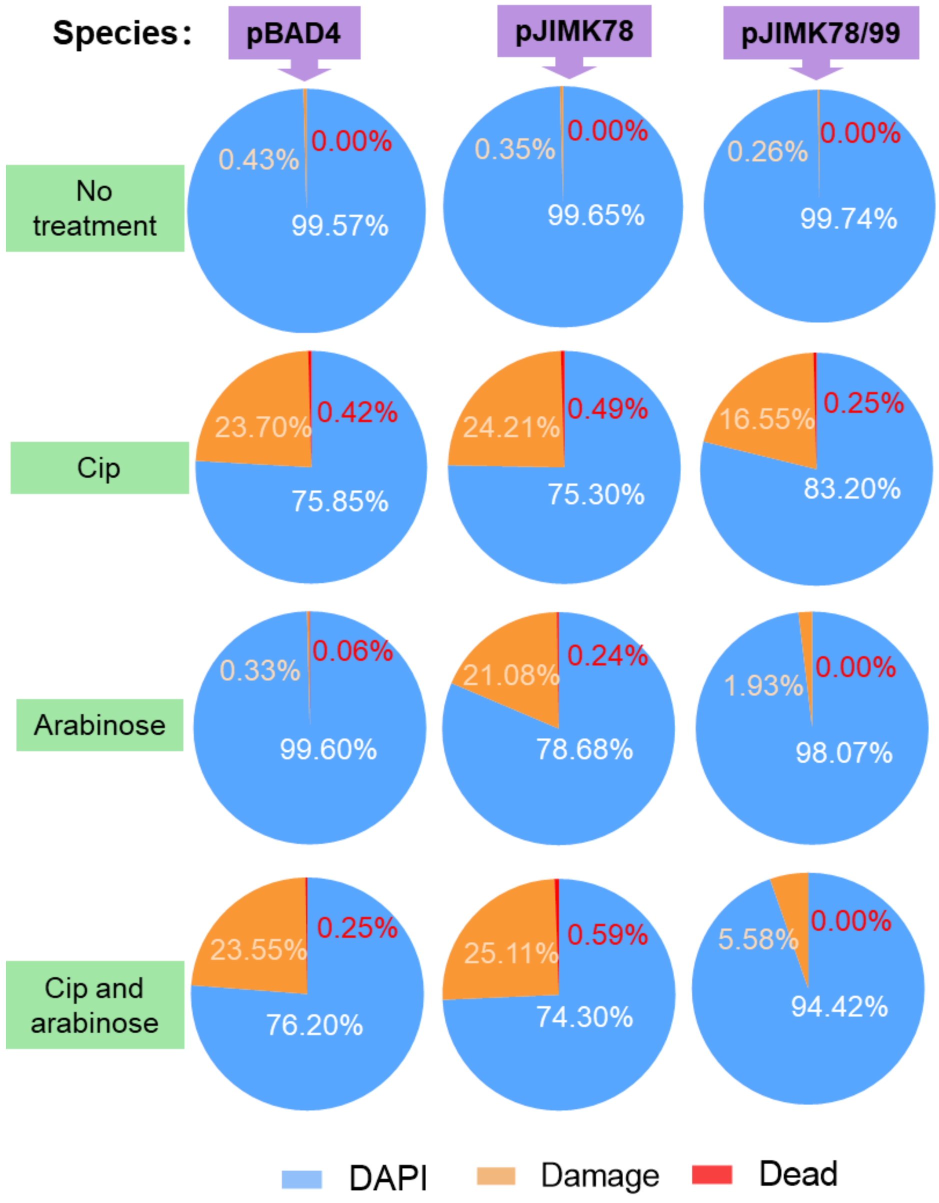

Figure S8. Fractions of DAPI- and/or PI-stained *E. coli* J53 strains that carried different plasmids (pBAD24, pJIMK78, and pJIMK78/99) under different treatment conditions. Antibiotic (Cip), arabinose (A), or both were combined to treat bacteria cells. All samples were tested in biological triplicate.

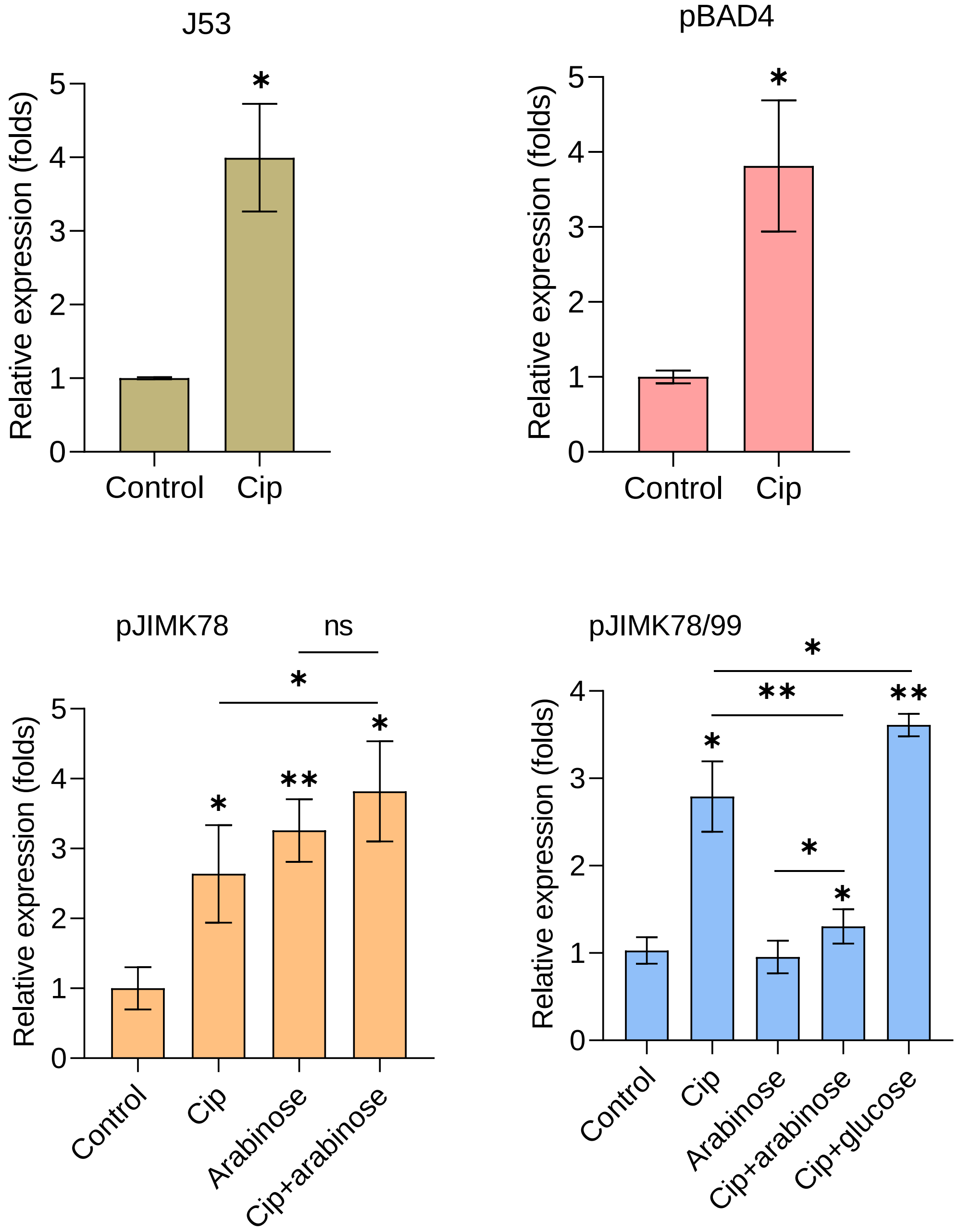

Figure S9. Relative expression of gene *sulA* in *E. coli* J53 carrying (**a**) no plasmids, (**b**) pBAD24, (**c**) pJIMK78, and (**d**) pJIMK78/99 under different conditions. Significant differences between the control and the treated groups were tested with Independent-sample *t* test and the Bonferroni correction, * $p < 0.05$ and ** $p < 0.01$.

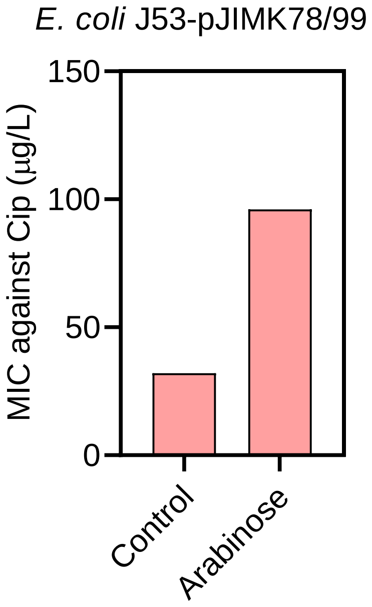

Figure S10. The MIC values of *E. coli* J53 carrying plasmid 78/99 against Cip (n = 3)

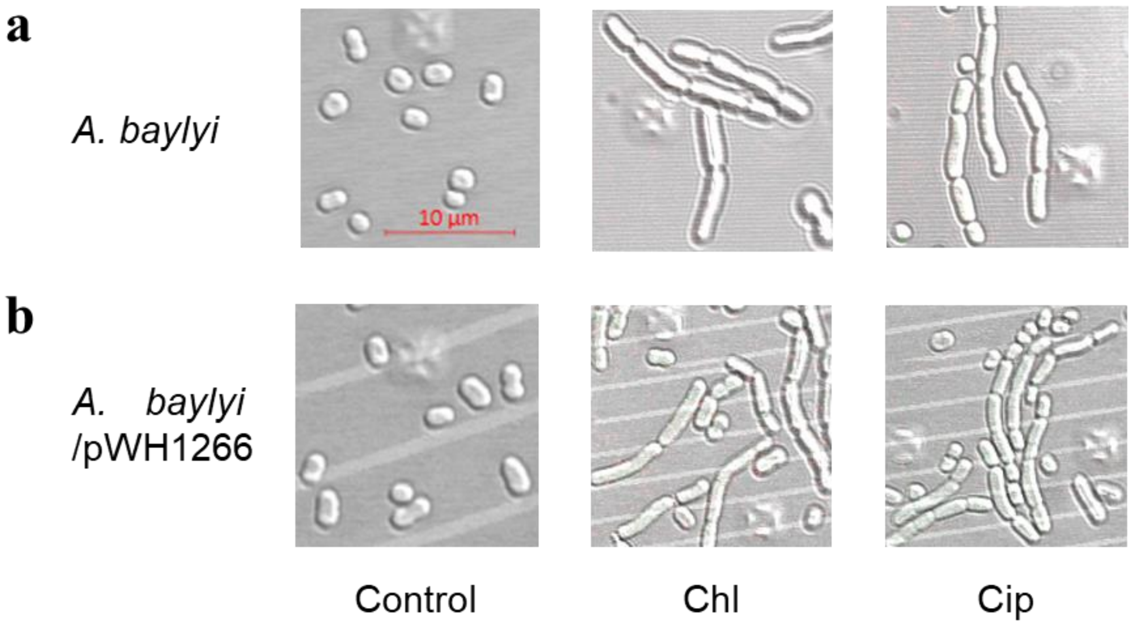

Figure S11. Morphological responses of (**a**) plasmid-free *Acinetobacter baylyi* (*A. baylyi*) ADP1 and (**b**) plasmid-bearing *A. baylyi*. The plasmid pWH1266 is non-mobile and has no TA system and no efflux pump. A sub-MIC of antibiotic chloramphenicol (Chl) or ciprofloxacin (Cip) was used to treat both types of bacterial cells.

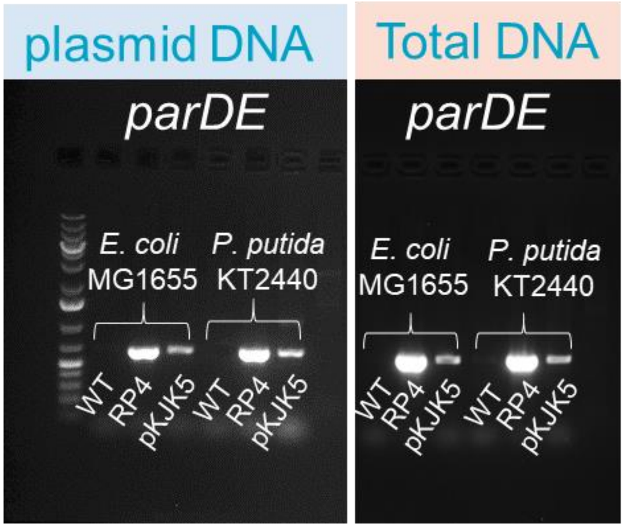

Figure S12. Gel imaging of toxin-antitoxin (TA) systems. Bands for *parDE* gene were only visualized in the extracted DNA from plasmid-bearing cells.

References

[1] Z. Yu, Y. Wang, J. Lu, P. L. Bond, J. Guo, *ISME J.* **2021**, *15* (7), 2117.

[2] M. Kamruzzaman, J. Iredell, *Sci. Rep.* **2019**, *9* (1), 1.

[3] A. Biran, H. B. Yoav, S. Yagur-Kroll, R. Pedahzur, S. Buchinger, Y. Shacham-Diamand, G. Reifferscheid, S. Belkin, *Anal. Bioanal. Chem.* **2011**, *400* (9), 3013.

[4] D. Hwang, Y. H. Lim, *FEMS Microbiol. Lett.* **2019**, *366* (4), fnz030.

[5] M. T. Suzuki, L. T. Taylor, E. F. DeLong, *Appl. Environ. Microbiol.* **2000**, *66* (11), 4605.
